# Childhood immunization and age-appropriate vaccinations in Indonesia

**DOI:** 10.1186/s12889-022-14408-x

**Published:** 2022-11-05

**Authors:** Hoi Chu, Anu Rammohan

**Affiliations:** Department of Economics and Australia Indonesia Centre, University of Western Australia, M251, 35 Stirling Highway, Perth, 6009 Australia

**Keywords:** Childhood immunization, Age-appropriate, Indonesia

## Abstract

**Background:**

Childhood immunization is a cost-effective way to protect individuals against communicable diseases. However, although there is a large literature on childhood immunization in Indonesia, there is a paucity of research on the age-appropriateness on measles and DTwP-3 vaccination, and the inequities in immunization coverage across Indonesia.

**Methods:**

In this paper, using seven waves of data from the nationally representative Indonesia Demographic and Health Surveys (DHS) covering the period 1991- 2017, we empirically analyse the socio-economic and demographic factors influencing the uptake of four routine vaccines (BCG, Polio-3, DTwP-3, and Measles). Specifically, using multivariate regression analysis, we identify the socio-economic and demographic factors influencing childhood immunization coverage. We further analyse the socio-economic and demographic correlates of the age-appropriateness of the measles and DTwP-3 vaccination coverage.

**Results:**

Our findings show that parental education and use of healthcare services are strong predictors of full immunization and age-appropriate vaccinations. This study also finds evidence of spatial heterogeneity in both full immunization rates and age-appropriate vaccinations for measles and DTwP-3 vaccines.

**Conclusions:**

Our analysis finds that despite an improvement in the timing of vaccinations over the last two decades, a significant proportion of children continue to receive their measles and DTwP vaccinations age inappropriately. In particular, we find that maternal education and maternal engagement with healthcare services are critical in improving age appropriateness of vaccinations. From a policy perspective, these results call for concerted efforts by policy makers to address regional gaps in access to health services and immunization coverage, as well as to improve the age-appropriateness of vaccination.

**Supplementary Information:**

The online version contains supplementary material available at 10.1186/s12889-022-14408-x.

## Introduction

Increasing accessibility to immunization is critical to achieving the Sustainable Development Goal 3 on Good Health and Well-being [1]. The effectiveness of childhood vaccination in averting deaths and improving health outcomes has been well documented in the literature [2–7]. Vaccinations are estimated to save approximately 2–3 million lives each year [8], and it has become a cornerstone of public health policy globally [9]. Vaccinations have contributed not only to the elimination of several preventable diseases in some advanced countries (including polio, diphtheria and pertussis), but also brought about multiple positive impacts on health outcomes in larger communities [3, 10, 11].

The World Health Organization’s (WHO) Expanded Program on Immunization (EPI) which began in 1974, aimed to reduce childhood morbidity and mortality. Despite this, immunization rates in many developing countries continue to be sub-optimal. Indonesia adopted the EPI in 1977, but in 2020 it was one of ten countries worldwide where more than 60% out of 23 million infants were unimmunized or only partially immunized with DTwP vaccine [12].

Recent research on childhood immunization in Indonesia [13, 14], used data from the Indonesian Demographic and Health Survey (IDHS) 2012 and found a strong association between complete vaccination and socio-economic factors. Other research from Indonesia has found evidence of religious [15] and rural–urban differences in measles vaccination coverage [16]; and showed that paternal education was an important predictor of child’s measles vaccination in six countries (including Indonesia) with the highest numbers of unvaccinated children [17]. The socio-economic factors influencing measles vaccination has also been studied in other developing countries including Nigeria, India, Bangladesh, and Nepal [18–22].

However, while the above literature has identified some of the socio-economic and cultural factors influencing childhood immunization, the issue of timing of vaccinations has not been studied in the Indonesian context. Vaccination efficacy (of measles and DTwP) against fatal and infectious diseases depends not only on being vaccinated, but also on the timing of vaccine administration [23, 24]. In the Indian context, previous research has shown that among children aged 12–23 months who were vaccinated against measles, only 30% received the measles vaccine at the appropriate age of 9 months, and only 31% received the DTwP-3 vaccine at the recommended age of 14 weeks [20].

The aim of this paper is to empirically examine the socio-economic and demographic factors influencing childhood immunization in Indonesia for four routine vaccines (BCG, Polio-3, DTwP-3, and Measles), and the age-appropriateness of measles and DTwP-3 vaccine administration. We use nationally representative data from seven rounds of the Indonesian Demographic Health Surveys, covering the period 1991–2017 to examine the socio-economic and demographic factors influencing trends in immunization rates over time.

Furthermore, we analyse if children were vaccinated for measles and DTwP-3 age-appropriately. Previous research has found that measles vaccination administered prior to 9 months of age may result in the neutralization of the vaccine by maternal antibodies [23, 24]. There is also evidence that seroconversion for measles is slightly lower among children who receive the first dose before or at 9 months of age (87% at 9 months, 95% at 12 months), because of persisting maternal antibodies [25]. Similarly, the efficacy of the DTwP vaccination also depends on appropriate timing. Pertussis, for example, is a highly contagious respiratory tract infection which may be life threatening in infants [26]. Age-appropriate vaccination against pertussis, starting with the first DTwP dose at 6 weeks, helps to minimize disease morbidity and mortality [27, 28]. Therefore, delays in administering the DTwP vaccine, leading to DTwP being given after the measles vaccine, or simultaneous DTwP and measles vaccinations at 9 months is associated with increased morbidity risk, especially for females [29, 30]. Previous research has found evidence of age-inappropriate vaccination among Indian children, with large gender differences observed in the propensity to immunize children age appropriately [19, 20]. To the best of our knowledge, the age-appropriateness of childhood immunization has not been studied in the Indonesian context.

Finally, there is large heterogeneity in vaccine coverage across different Indonesian provinces. Therefore, from a policy perspective it is critical to identify the key socio-economic and demographic drivers of vaccination, and the factors influencing the timing of vaccinations, particularly for measles and DTwP-3.

## Methods

The data for this analysis come from seven waves of the nationally representative Indonesian Demographic Health Surveys (IDHS) conducted in the years 1991, 1994, 1997, 2002, 2007, 2012, and 2017. The IDHS is part of the worldwide Demographic and Health Surveys which is a large multi-topic survey focusing on maternal and child health. The IHDS is fielded by the Indonesian National Family Planning Coordinating Board in collaboration with other governmental institutions including the Ministry of Health and Statistics Indonesia. The surveys use a consistent questionnaire in every survey which makes it possible to compare children across various years. The dataset is publicly available and can be obtained free of cost upon registration from the DHS website (https://dhsprogram.com/).

The IDHS is a cross-sectional survey that focuses on women aged 15–49 years and their households. The data on immunization comes from the Women's Questionnaire, which contains detailed information on the immunization history of the last two children. The survey also collects data from ever-married women on socio-economic and demographic characteristics of households and household members, including information on children and maternal health outcomes, age, education, occupation status, living condition, and economic status. We combine this information with our detailed mother-level responses to the child immunization questions. A child’s vaccination status is verified by their mother’s report or a vaccination card which is sighted by the enumerator. Our final sample consists of a nationally representative sample of 73,714 children aged 12–59 months old at the time of survey.

### Measures of vaccination

#### Immunization status of children

The aim of our analysis is to examine the socio-economic and demographic factors associated with a child’s immunization status. To this end, our outcome variables include the immunization status of a child aged 12–59 months for the four following vaccines: BCG, DTwP-3, Polio-3, and Measles. Specifically, respondents were asked: (i) Has (name) ever received a BCG vaccination against tuberculosis, that is, an injection in the arm or shoulder that usually causes a scar?, (ii) How many times did (name) receive the oral polio vaccine?, (iii) How many times did (name) receive the DTwP vaccine?, (iv) Has (name) ever received a measles vaccination, that is, an injection in the arm to prevent measles?

Note that our sample of 73,390 eligible children include vaccination responses based on either the mother’s response or verified by vaccination cards. The children’s vaccination status differed across the four different vaccinations. Specifically, for BCG vaccinations, 17.45% did not receive the vaccinations, and among vaccinated children, the vaccination status was verified by vaccination cards for 16,314 children (22.13%) and by mother’s responses for 44,216 children (59.98%). For DTwP-3, these figures were 14,858 (20.16%) for vaccination cards and 32,432 (44.00%) via mother’s recall. Similarly, for Polio-3, the vaccinated children included 14,104 (20.49%) based on vaccination cards and 35,826 (48.6%) based on mother’s responses. Finally for measles, 14,256 children (19.34%) had vaccination cards and another 39,761 (53.94%) were based on mother’s response. We observe that each of the vaccinations, the mother’s responses had a larger sample. Therefore, to increase the representativeness of the sample in our analysis we use responses based on either mother’s recall or vaccination card.^1^ Accordingly, a child is classified as being: (i) fully immunized if they received all four vaccines; (ii) partially immunized if they have received at least some vaccinations; and finally (iii) Unvaccinated if they did not receive any of the above vaccines.

**Table 1:** Descriptive statistics of key variables

Background characteristics	Immunization status		
	No (%)	Partial (%)	Full (%)
Child is female	14.27	28.17	57.57
Child is male	14.39	28.81	56.80
Child’s age (in months)	34.21	33.19	33.41
Child’s birth-order 1^st^	10.58	27.40	62.02
Child’s birth order: 2^nd^	11.04	26.03	62.93
Child’s birth-order: 3^rd^	19.60	31.05	49.35
**Mother’s age**			
*Mother’s age: 15–19*	19.77	37.12	43.11
*Mother’s age: 20–29*	13.43	28.80	57.78
*Mother’s age: 30* +	14.98	27.73	57.29
**Mother’s education**			
*No education*	38.80	31.35	29.85
*Primary*	18.45	32.15	49.40
*Secondary*	6.07	25.01	68.92
*Higher*	3.73	17.51	78.76
Number of under 5 children	1.55	1.36	1.30
Media access: no	35.28	29.15	35.57
Media access: yes	11.61	28.41	59.99
**Mother’s use of maternal healthcare services**			
*Home delivery*	19.84	31.87	48.29
*Institutional delivery*	5.37	23.00	71.63
*Mother had 3 or fewer ANC visits*	35.77	32.99	31.24
*Mother had 4* + *ANC visits*	7.59	27.08	65.34
*Mother used any PNC/ANC services: no*	54.00	25.91	20.09
*Mother used any PNC/ANC services: yes*	9.88	28.78	61.34
**Father’s education**			
*No education*	37.63	30.20	32.18
*Primary*	19.50	31.92	48.58
*Secondary*	7.71	26.23	66.06
*Higher*	3.50	18.28	78.22
**Mother's occupation**			
*Not working*	13.61	28.73	57.66
*Professional*	4.64	19.61	75.75
*Agricultural*	23.92	31.65	44.43
*Industrial*	11.46	27.92	60.62
*Clerical, sales, services*	9.20	26.65	64.15
**Father's occupation**			
*Not working*	16.60	30.03	53.37
*Professional*	5.10	21.70	73.20
*Agricultural*	22.40	30.88	46.72
*Industrial*	10.33	28.99	60.68
*Clerical, sales, services*	9.83	26.64	63.53
Household headed by a female	14.00	30.40	55.60
Household headed by a male	14.35	28.38	57.27
Rural household	18.41	30.00	51.59
Urban household	7.62	26.01	66.37
**Wealth quintile**			
*Poorest*	22.90	31.38	45.72
*Poorer*	17.27	30.55	52.17
*Middle*	14.07	28.81	57.12
*Richer*	9.97	27.46	62.57
*Richest*	6.38	23.77	69.85
**Number of observations**	**11,309**	**19,890**	**42,515**

The table presents the proportion of vaccinated children by vaccination cards and mother’s recall

Source: Authors’ calculations based on Indonesia DHS 1991–2017

**Fig. 1:**
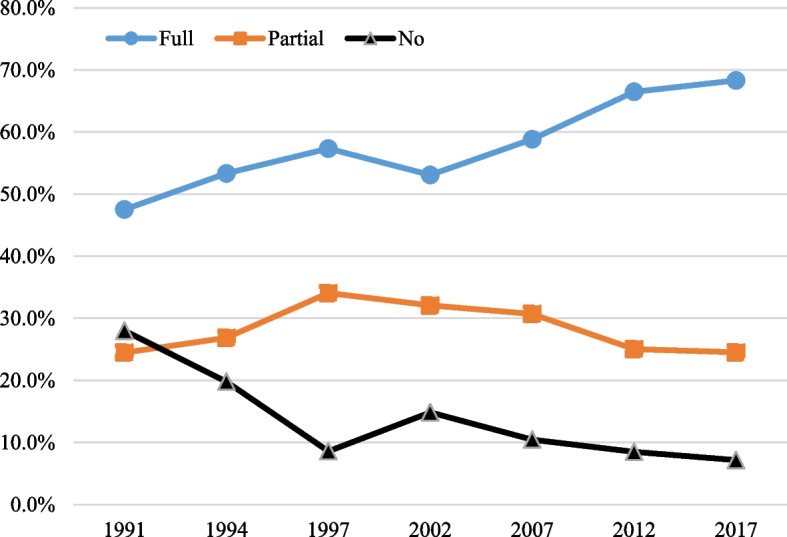
Immunization coverage over 1991–2017. Source: Authors’ calculations based on Indonesia DHS 1991–2017

Figure 1 presents aggregate trends in vaccination coverage over the period 1991–2017. We observe a steady improvement in vaccination rates over time, with the proportion of fully-immunized children increasing from just under 50% in 1991 to 70% in 2017, with a decrease in the partial immunization. There is also a corresponding decrease in the proportion of un-vaccinated children from around 30% in 1991 to less than 10% in 2017.

#### Age-appropriateness of vaccination

Next, we investigate the age-appropriateness of the timing of the DTwP-3 and measles vaccinations for the sub-sample of vaccinated children, whose vaccination cards were sighted by the interviewers. As information on the timing of vaccination is only available for those children with a Vaccination Card, the analysis on the timing of the vaccination is restricted to only those children with a Vaccination Card.

In Indonesia, the DTwP-3 and measles are scheduled to be administered when a child is 4 and 9 months old, respectively [31].^2^ Accordingly, we construct two measures: (i) *Age appropriateness for DTwP-3* = 1 if a child received their DTwP-3 vaccine at age 4–5 months, 0 otherwise; and (ii) *Age-appropriateness for measles* = 1 if the child received their measles vaccination at age 9–10 months, 0 otherwise.

**Fig. 2:**
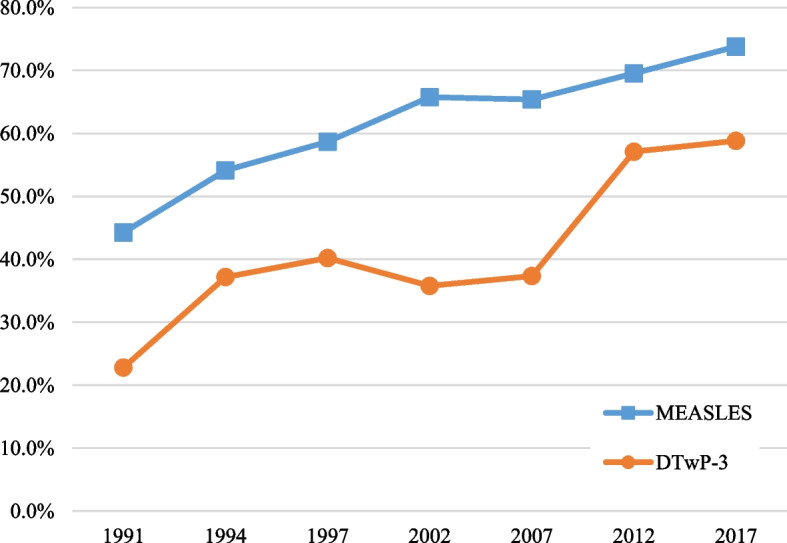
Proportion of age-appropriate vaccinations: Measles and DTwP-3. Source: Authors’ calculations based on Indonesia DHS 1991–2017

The trends in the timing of the DTwP-3 and measles vaccination are presented in Fig. 2. As previously mentioned, the efficacy of vaccinations is contingent on the timing of the vaccination, and it is critical that the vaccines are administered age-appropriately.

Although there have been large improvements in the age-appropriateness of measles and DTwP-3 vaccinations, a high proportion of children continue to be age-inappropriately vaccinated. Specifically, in 1991 only around 41% and 22% of the eligible vaccinated children had been vaccinated age-appropriately for measles and DTwP-3, respectively. These figures have increased over time, and by 2017 we observe that nearly 70% (58%) of the vaccinated children have been administered measles (DTwP-3) vaccinations age-appropriately.

### Explanatory variables

The main explanatory variables in our analysis include the socio-economic and demographic characteristics of the child’s household, access to health care facilities and geographical factors. More specifically, we include the child’s sex, age, and birth order; maternal characteristics such as mother’s age, education level, mother’s use of healthcare services, and father’s education level. We also include the socio-economic and demographic characteristics of the child’s household such as their province of residence, urban or rural residence, and the household head’s sex. The household’s economic status is measured using the wealth index which is available in the dataset. The wealth index is constructed using PCA (Principal Component Analysis) for all the assets owned by the household. The entire distribution is divided into five wealth quintiles. As the pre-constructed wealth index was not available in the 1991 and 1994 survey rounds, we used the PCA method to construct 5 wealth quintiles for these two years, using information on household assets.

### Estimation strategy

#### Child’s immunization status: ordered probit model

We begin by considering the associations between a child’s immunization status and an array of socio-economic and demographic characteristics. Our main variable of interest is estimated using Equation 1 below:

1 $${\mathrm{Immunization}}_{j,p,t}= {\beta }_{0}+{\beta }_{1}{ ch}_{j,p,t}+{\beta }_{j} {hh}_{j,p,t}+{\theta }_{p}+{\mu }_{t}+{\varepsilon }_{j,t}$$

where, ${\mathrm{Immunization}}_{j,p,t}$ refers to the latent immunization status of child-*j* in province *p* at time *t*. The term ${ch}_{j,p,t}$ includes a vector of child characteristics such as their sex, age and birth order. The term ${hh}_{j,p,t}$ includes variables relating to the socio-economic characteristics of the child’s household, and the characteristics of the child’s mother and father.

Our outcome variable is a measure of the child’s immunization status, which can potentially fall into three categories, and has a natural ordering. Accordingly, we estimate an Ordered Probit model where the dependent variable, a child’s immunization status can be classified into three categories: no immunization (0), partial immunization (1) and full immunization (2). Each of these discrete categories of the dependent variable can be explained by the same set of explanatory variables.

### Age-appropriateness of vaccinations: Probit model

Next, we analyze the age-appropriateness of the measles and DTwP vaccinations, using the sub-sample of 27,500 children with vaccination cards. Within this sample, a sub-sample of 13,661 children received the measles vaccine and 14,199 children received the DPT3 vaccine that also provide information on the timing of the vaccination. Using these two sub-samples of vaccinated children, we create a binary variable to indicate if the child has received immunization at the appropriate age or not, and estimate the following equation.

2 $$Probit\{{A}_{j,p,t}=1|control\}= {\beta }_{0}+{\beta }_{j}{ ch}_{j,p,t}+{\beta }_{k} {hh}_{j,p,t}+{\theta }_{p}+{\mu }_{t}+{\varepsilon }_{j,p,t}$$

where ${A}_{j,p,t}$ is a binary variable, taking a value of 1 if a child received the measles vaccine or DTwP vaccine at the appropriate age, and 0 otherwise. Premature or delayed vaccination are regarded as not being age-appropriately vaccinated.

Note that in both estimations we include indicator variables for provinces to account for unobserved heterogeneity (represented by parameter ${\theta }_{p}$). We also add indicator variables for survey years ($\mathrm{represented by} {\mu }_{t})$ to control for changes in immunization levels over time.

## Results

Descriptive statistics of the main variables used in empirical analysis are presented in Table 1. We disaggregate the sample of 73,390 children aged 12–59 months by their immunization status. Over the period 1991–2017, we observe that approximately 57.18% of the sample were fully immunized, 28.49% were partially immunized and 14.33% did not receive any vaccinations. Older children were less likely to be vaccinated, indicating improvements in immunization over time.

Similarly, fully immunized children were more likely to have younger and better educated mothers, while un-vaccinated children had less educated and older mothers. We observe similar trends with father’s education.

Mother’s use of healthcare services or exposure to health professionals is strongly associated with childhood immunization. For example, around 71.63% of children who were delivered institutionally were fully immunized, much higher than the cohort of those delivered at home (48.29%). Likewise, 65.34% and 61.34% of children was fully vaccinated if their mothers had 4 or more antenatal care (ANC) visits and used any PNC/ANC (prenatal/antenatal) healthcare service,^3^ respectively. The rates of complete vaccination for children whose mothers had 3 or fewer ANC visits and used none of PNC/ANC services are 31.24% and 20.09%, respectively. On the other hand, among un-vaccinated children, only 5.37% had institutional deliveries, only 7.59% of their mothers had 4 + ANC visits, and only 9.88% had used any PNC/ANC services. This suggests that the greater availability and accessibility to healthcare services and/or health workers has the potential to improve vaccination coverage.

Finally, we observe that full immunization rates increase monotonically with each higher wealth quintile. Specifically, the proportion of children that are fully vaccinated increased from 45.72% in the poorest quintile to nearly 70% among children in the richest wealth quintile. On the other hand, the proportion of unvaccinated children decreases from 22.9% among children in the poorest wealth quintile to only 6.38% among children in the richest wealth quintile.

### Regional variations in immunization status

**Fig. 3:**
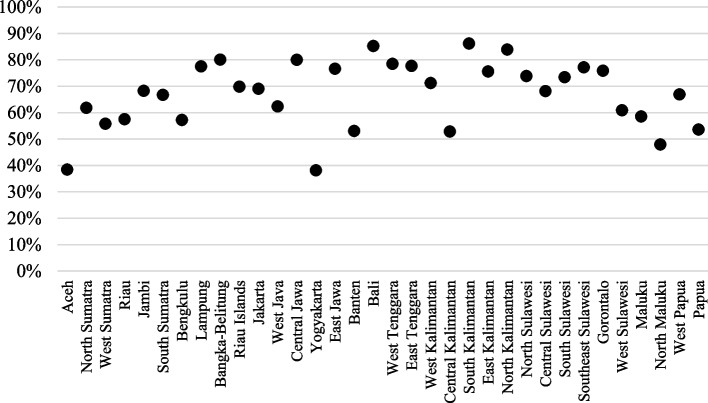
Full immunization rate by provinces 2017. Source: Authors’ calculations based on Indonesia DHS 2017

We observe large regional differences in aggregate vaccination coverage at the provincial level (Fig. 3). In 2017, only 5 out of 34 provinces had full immunization rates above 80%, while 11 provinces had full immunization rates below 60%.

**Fig. 4:**
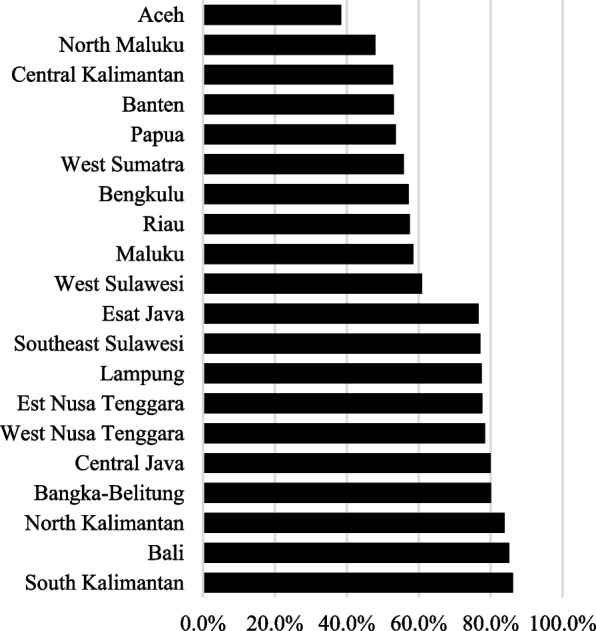
Provinces with the highest and lowest rates of full immunization in 2017. Source: Authors’ calculations based on Indonesia DHS 2017

Figure 4 presents the 10 provinces with the highest and lowest full immunization rates in 2017. The highest rates of full immunization were in the provinces of Bali (86.2%) and South Kalimantan (85.2%), while Aceh (38.4%) had the lowest rate. According to Indonesia’s Statistical Yearbook [32], poverty rates in September 2019 in Bali, South Kalimantan, and Aceh were 3.61%, 4.47%, and 15.01%, respectively. This further shows that economic factors may be influencing vaccine accessibility.

**Fig. 5:**
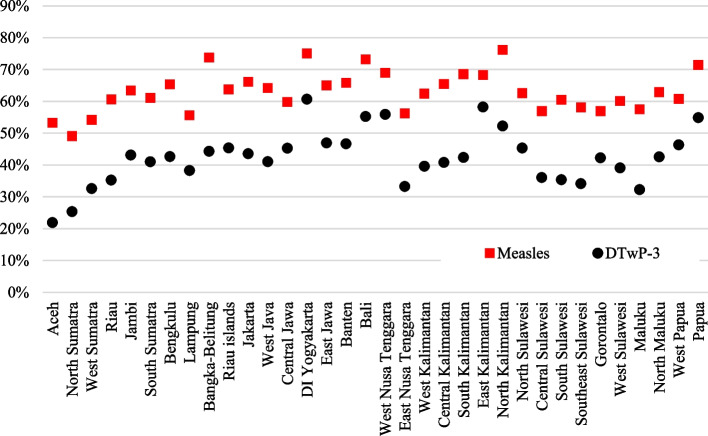
Age-appropriateness of measles and DTwP-3 vaccinations. > Source: Authors’ calculations based on Indonesia DHS 1991–2017

Similarly, there are provincial variations in the age-appropriateness of vaccinations for measles and DTwP-3 (Fig. 5). Generally, measles vaccine was administered more age-appropriately than the DTwP-3 vaccine. With regards to DTwP-3, it is concerning that the age-appropriate vaccination rates were below 50% in 28 of the 34 Indonesian provinces.

**Fig. 6:**
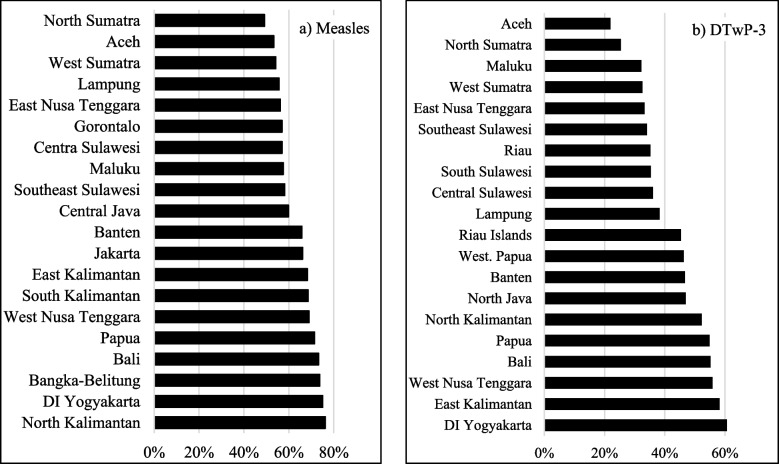
Top and bottom provinces with age-appropriate vaccination. Source: Authors’ calculations based on Indonesia DHS 1991–2017

Figure 6 shows the top and bottom 10 provinces in terms of age-appropriate vaccinations for measles (left panel) and DTwP-3 (right panel). In particular, we again find that Aceh has low rates of age-appropriate vaccinations, with around 53% and 22% of children age-appropriately vaccinated with measles and DTwP-3, respectively.

### Empirical results

The main results of our empirical analysis are presented in Tables 2 and 3. Table 2 presents the Ordered Probit marginal effects for the child’s immunization status, while Table 3 presents the Probit estimation results for the age-appropriateness of the administration of the measles and DTwP-3 vaccinations, respectively.

**Table 2:** Ordered Probit estimates of immunization status

	(1)	(2)	(3)
	No	Partial	Full
Child is male	0.002 (0.002)	0.001 (0.001)	-0.003 (0.003)
Child’s age (in months)	-0.002^***^ (0.000)	-0.002^***^ (0.000)	0.004^***^ (0.001)
Child’s age (in months) squared	0.000^***^ (0.000)	0.000^***^ (0.000)	-0.000^***^ (0.000)
**Birth order (ref: 1**^**st**^** birth)**			
*2*^*nd*^* birth*	-0.004 (0.002)	-0.003 (0.002)	0.007 (0.004)
*3*^*rd*^ + *birth*	0.018^***^ (0.003)	0.013^***^ (0.002)	-0.031^***^ (0.005)
**Mother’s age**			
*Age: 20–29*	-0.025^***^ (0.006)	-0.018^***^ (0.004)	0.043^***^ (0.010)
*Age: 30* +	-0.035^***^ (0.006)	-0.026^***^ (0.004)	0.061^***^ (0.010)
**Mother’s education (ref: no education)**			
*Primary*	-0.037^***^ (0.004)	-0.026^***^ (0.003)	0.063^***^ (0.006)
*Secondary*	-0.080^***^ (0.004)	-0.058^***^ (0.003)	0.138^***^ (0.007)
*Higher*	-0.085^***^ (0.006)	-0.061^***^ (0.005)	0.146^***^ (0.011)
Access to media	-0.034^***^ (0.003)	-0.025^***^ (0.002)	0.059^***^ (0.005)
Place of delivery: institutional	-0.033^***^ (0.002)	-0.024^***^ (0.002)	0.056^***^ (0.004)
Has 4 + ANC visits	-0.080^***^ (0.002)	-0.057^***^ (0.002)	0.137^***^ (0.004)
Place of ANC-PNC: hospital/health worker	-0.118^***^ (0.003)	-0.085^***^ (0.002)	0.203^***^ (0.005)
**Father’s education (ref: no education)**			
*Primary*	-0.024^***^ (0.005)	-0.017^***^ (0.003)	0.041^***^ (0.008)
*Secondary*	-0.050^***^ (0.005)	-0.036^***^ (0.003)	0.086^***^ (0.008)
*Higher*	-0.066^***^ (0.006)	-0.047^***^ (0.005)	0.113^***^ (0.011)
Household head is male	-0.013^***^ (0.004)	-0.010^***^ (0.003)	0.023^***^ (0.007)
Urban	0.004^*^ (0.002)	0.003^*^ (0.002)	-0.008^*^ (0.004)
Number of children under 5	0.020^***^ (0.001)	0.014^***^ (0.001)	-0.034^***^ (0.002)
**Wealth index (ref: poorest)**			
*poorer*	-0.006^**^ (0.003)	-0.004^**^ (0.002)	0.011^**^ (0.004)
*middle*	-0.015^***^ (0.003)	-0.011^***^ (0.002)	0.026^***^ (0.005)
*richer*	-0.023^***^ (0.003)	-0.017^***^ (0.002)	0.040^***^ (0.005)
*richest*	-0.032^***^ (0.003)	-0.023^***^ (0.002)	0.055^***^ (0.006)
**Mother’s occupation (ref: not working)**			
*Professional job*	-0.009^*^ (0.005)	-0.007^*^ (0.004)	0.016^*^ (0.009)
*Agricultural sector*	-0.008^***^ (0.003)	-0.006^***^ (0.002)	0.014^***^ (0.004)
*Industrial sector*	-0.006 (0.004)	-0.004 (0.003)	0.011 (0.007)
*Clerical, sales and services*	-0.002 (0.002)	-0.001 (0.002)	0.003 (0.004)
**Father’s occupation (ref: not working)**			
*Professional job*	-0.014^***^ (0.005)	-0.010^***^ (0.004)	0.024^***^ (0.009)
*Agricultural sector*	0.001 (0.004)	0.001 (0.003)	-0.002 (0.007)
*Industrial sector*	-0.004 (0.004)	-0.003 (0.003)	0.008 (0.007)
*Clerical, sales and services*	0.000 (0.004)	0.000 (0.003)	-0.000 (0.007)
**Year (ref: 1991)**			
*1994*	-0.046^***^ (0.003)	-0.032^***^ (0.002)	0.078^***^ (0.006)
*1997*	-0.074^***^ (0.003)	-0.059^***^ (0.002)	0.133^***^ (0.005)
*2002*	-0.005 (0.004)	-0.003 (0.002)	0.008 (0.006)
*2007*	0.003 (0.004)	0.002 (0.002)	-0.004 (0.006)
*2012*	-0.020^***^ (0.004)	-0.013^***^ (0.003)	0.032^***^ (0.007)
*2017*	-0.024^***^ (0.005)	-0.015^***^ (0.003)	0.039^***^ (0.008)
Province Dummies	Yes	Yes	Yes
***N***	**73,714**	**73,714**	**73,714**

Note: Results are based on full sample; marginal effects are reported; robust standard errors in parentheses; ^*^p < 0.10, ^**^p < 0.05, ^***^p < 0.01

**Table 3:** Probit estimates of Age-appropriate vaccination among vaccinated children

	(1)	(2)
	Measles	DTwP-3
Child is male	-0.010 (0.008)	-0.006 (0.008)
Child’s age (in months)	-0.007^***^ (0.002)	-0.002 (0.002)
Child’s age (in months) squared	0.000^***^ (0.000)	0.000 (0.000)
**Birth order (ref: 1**^**st**^** birth)**		
*2*^*nd*^* birth*	-0.015 (0.011)	0.019^*^ (0.010)
*3*^*rd*^ + *birth*	-0.027^**^ (0.013)	-0.022^*^ (0.013)
**Mother’s age**		
*Age: 20–29*	0.028 (0.026)	-0.007 (0.027)
*Age: 30* +	0.040 (0.028)	-0.001 (0.028)
**Mother’s education (ref: no education)**		
*Primary*	0.063^***^ (0.024)	0.038 (0.025)
*Secondary*	0.105^***^ (0.025)	0.092^***^ (0.026)
*Higher*	0.100^***^ (0.030)	0.084^***^ (0.031)
Access to media	0.045^***^ (0.017)	0.044^**^ (0.018)
Place of delivery: institutional	0.045^***^ (0.010)	0.055^***^ (0.010)
Has 4 + ANC visits	0.046^***^ (0.013)	0.027^**^ (0.014)
Place of ANC-PNC: hospital/health worker	0.058^**^ (0.023)	0.033 (0.025)
**Father’s education (ref: no education)**		
*Primary*	0.007 (0.028)	0.008 (0.030)
*Secondary*	0.024 (0.029)	0.023 (0.031)
*Higher*	0.046 (0.033)	0.022 (0.034)
Household head is male	0.005 (0.018)	0.006 (0.018)
Urban	0.011 (0.010)	0.026^***^ (0.010)
Number of children under 5	-0.009 (0.007)	-0.032^***^ (0.007)
**Wealth index (ref: poorest)**		
*poorer*	0.007 (0.013)	0.011 (0.013)
*middle*	0.020 (0.013)	0.025^*^ (0.013)
*richer*	0.019 (0.014)	0.024^*^ (0.014)
*richest*	0.042^***^ (0.015)	0.031^**^ (0.015)
**Year (ref: 1991)**		
*1994*	0.096^***^ (0.016)	0.170^***^ (0.015)
*1997*	0.136^***^ (0.017)	0.185^***^ (0.016)
*2002*	0.174^***^ (0.018)	0.126^***^ (0.016)
*2007*	0.134^***^ (0.018)	0.106^***^ (0.016)
*2012*	0.216^***^ (0.017)	0.253^***^ (0.016)
*2017*	0.214^***^ (0.018)	0.277^***^ (0.017)
Province Dummies	Yes	Yes
*N*	13,860	14,531

The sample only includes vaccinated children whose vaccination cards were sighted by the enumerators; marginal effects are reported; robust standard errors are in brackets, ^*^p < 0.10, ^**^p < 0.05, ^***^p < 0.01

### Role of socio-economic factors in influencing immunization status of children

Our results show that mother’s age and educational attainment are significantly associated with a child’s immunization status. In particular, relative to a child with a younger mother (aged 19 years or below), a child has a 4.3 and 6.1 percentage points higher probability of being fully immunized if the mother was aged 20–29 years and 30 years or higher, respectively.

Similarly, relative to a mother with no education, each higher level of education is associated with a statistically significant and higher probability of being fully immunized, and a negative probability of being unvaccinated. We observe similar trends with regards to father’s education.

Not surprisingly, the three measures of engagement with health care services (measured using institutional place of delivery, four ANC visits and use of ANC/PNC) are all statistically significant and positively signed with a child being fully immunized, and negatively correlated with a child being unvaccinated. Specifically, a child’s institutional delivery is associated with a 5.6 percentage points higher probability of being fully immunized, compared to children who had home births. Similarly, mother’s use of antenatal care and/or prenatal care is associated with a 20.3 percentage points higher probability of a child being fully immunized. Mother’s access to media is also positively associated with full immunization.

In terms of economic status, not surprisingly, our estimates show that relative to children from the lowest wealth quintile, each higher wealth quintile is associated with a higher probability that the child is fully immunized. We further observe that the probability of being fully immunized is 3.4 percentage points lower if there are other children in the household aged under-5 years.

### Age appropriateness of vaccination

In Table 3 we present the estimation results for the age appropriateness of the measles vaccination (Column 1) and DTwP-3 (Column 2), among vaccinated children whose vaccination cards were sighted by the enumerators.

Our results show that mother’s education is statistically significant and positively associated with the child receiving both the measles and the DTwP-3 vaccinations at the appropriate age. In particular, relative to no education, Secondary and Higher than secondary level of education increases the probability of that the child has received measles and DTwP-3 vaccinations at the appropriate age by 10 percentage points and 8.4 percentage points, respectively. Father’s education on the other hand is not statistically significant.

As with immunization status, engagement with health care services, through institutional delivery and having more than four ANC visits is associated with a higher probability that the child has received both measles and DTwP-3 vaccinations age appropriately.

While urban location is positively associated with receiving DTwP-3 vaccinations age-appropriately, it is not statistically significant for measles. Higher birth order children have a lower probability of being vaccinated age appropriately.

Finally, the probability of a child receiving vaccinations age appropriately has improved monotonically over time. Specifically, relative to 1991, the probability that a child was vaccinated at the appropriate age was 21.4 and 27.7 percentage points higher in 2017 for measles and DTwP-3, respectively.

## Discussion

Childhood immunization is among the most effective ways to protect individuals against communicable diseases. However, despite efforts to increase the immunization rates among children, they remain sub-optimal in many developing countries.

In this paper we analyzed the socio-economic and demographic factors influencing childhood immunization, and the age-appropriateness of measles and DTwP vaccinations in Indonesia, using data from seven waves of the Indonesian Demographic Health Survey (IDHS) covering the period 1991–2017. Our study shows that despite progress in improving childhood immunization, by 2017 over 20% of Indonesian children continued to remain unvaccinated or were only partly immunized. Moreover, we observe large heterogeneity across geographical regions and by household wealth status.

Our results are by and large in keeping with previous studies on vaccination coverage from Indonesia [13–17] that also find urban residence and parental education as being associated with childhood immunization. However, these studies were single year studies. Our finding of a positive association between household wealth and full immunization of children is consistent with previous research from Indonesia [34], but contrasts with the findings of Efendi et al. [13] who use only IDHS 2012.

One of the strengths of our study is that we are the first to examine the age-appropriateness of the timing of the measles and DTwP vaccinations in the Indonesian context. We find that maternal education and maternal engagement with healthcare services are critical in improving age appropriateness of vaccinations. This finding is in keeping with previous research from Senegal [33]. We also find large provincial-level differences in both full immunization as well as in the age-appropriateness of measles and DTwP vaccinations.

This study suffers from a couple of limitations. Firstly, relative to earlier rounds of the survey, there is greater availability of vaccination cards in more recent surveys. Subsequently, our analysis on age-appropriate vaccinations may over-represent more recent cohorts. Secondly, the data used in the analysis are repeated cross-section, so we are unable to infer causality. Therefore, our analysis can only identify associations between childhood vaccinations and socio-economic factors. Thirdly, access to health care facilities was a critical factor influencing vaccination uptake. However, we are unable to observe distance to health care facilities. Finally, our sample only includes only data until 2017. However, Indonesian vaccination surveillance data indicates that national DTwP-3 vaccination rates have declined in the pandemic period – 77% in 2020 and 67% in 2021. Our analysis is unable to capture these changes as the last available DHS survey in Indonesia is the 2017 survey.

## Conclusions

Our analysis finds that although the timing of vaccinations has improved over time, a significant proportion of children continue to receive their measles and DTwP vaccinations age inappropriately. Given the low immunization coverage in some provinces, the much lower rates of age-appropriate vaccinations in a number of provinces raise questions about the real efficacy of vaccines in protecting against communicable diseases.

From a policy perspective, our study highlights the critical role of engagement with health services and parental education in both improving immunization rates as well as the age-appropriateness of the vaccinations. The wide regional differences observed in vaccination coverage across Indonesia’s provinces also require concerted efforts by policy makers to address regional gaps in immunization coverage, as well as to improve the age-appropriateness of vaccination.

## Supplementary Information

**Additional file 1.**

## Data Availability

The dataset is publicly available and can be obtained free of cost upon registration from the following website: https://dhsprogram.com/

## Footnotes

1 Table A1 in the Appendix presents information on vaccination status for all 7 rounds of the IDHS, based on whether the information came from mother’s recall or vaccination card.

2 For comparison purposes, Figure A1 in the Supplementary Appendix presents vaccination rates for DTwP dose 1,2 and 3, and Figure A2 in the Appendix presents the age-appropriateness of DTwP-2 and DTwP-3 vaccines.

3 This refers to those who were exposed to public/private hospitals, health centers, or had interacted with health professionals.
