# Supplementary material for: Childhood immunization and age-appropriate vaccinations in Indonesia
Source: BMC Public Health. 2022 Nov 5;22:2023. doi: 10.1186/s12889-022-14408-x (PMC9636708; doi:10.1186/s12889-022-14408-x)
Supplement: Supplementary file 1 — Additional file 1. [file 12889_2022_14408_MOESM1_ESM.docx]

**SUPPLEMENTARY APPENDIX**

**Table A1**. Vaccination rates by vaccine type and survey years

| Year | BCG | | DTwP-3 | | Polio-3 | | Measles | |
| --- | --- | --- | --- | --- | --- | --- | --- | --- |
|  | Vacc. card | Mother’s report | Vacc. card | Mother's report | Vacc. card | Mother's report | Vacc. card | Mother's report |
| 1991 | 20.2 | 50.5 | 17.9 | 35.0 | 18.0 | 35.0 | 16.9 | 40.8 |
| 1994 | 22.0 | 56.3 | 20.1 | 39.9 | 20.2 | 41.1 | 18.8 | 48.0 |
| 1997 | 17.9 | 67.5 | 16.0 | 48.9 | 16.1 | 61.8 | 15.1 | 60.8 |
| 2002 | 19.2 | 63.1 | 17.0 | 42.0 | 18.1 | 49.1 | 16.4 | 57.8 |
| 2007 | 22.4 | 62.4 | 20.2 | 44.8 | 20.9 | 55.2 | 20.0 | 58.9 |
| 2012 | 27.5 | 62.3 | 26.1 | 45.8 | 26.8 | 50.9 | 25.2 | 58.3 |
| 2017 | 50.1 | 41.6 | 46.7 | 32.5 | 45.4 | 29.6 | 44.8 | 37.6 |

Notes: The table presents of the proportion of vaccinated children by vaccination cards and mother’s recall.

Source: Authors’ calculations based on Indonesia DHD 1991-2017

**Figure A1**. DTwP-1, DTwP-2, and DTwP-3 vaccinations by survey years

Source: Authors’ calculations based on Indonesia DHD 1991-2017

**Figure A2**. Age-appropriateness of DTwP-2 and DTwP-3 vaccinations by survey years

Source: Authors’ calculations based on Indonesia DHD 1991-2017
